# Epigenetic Alterations in Prescription Opioid Misuse: New Strategies for Precision Pain Management

**DOI:** 10.3390/genes12081226

**Published:** 2021-08-10

**Authors:** Maria Carla Gerra, Cristina Dallabona, Lars Arendt-Nielsen

**Affiliations:** 1 Center for Neuroplasticity and Pain (CNAP), SMI^®^, Department of Health Science and Technology, Aalborg University, 9220 Aalborg, Denmark; LAN@hst.aau.dk; 2 Department of Chemistry, Life Sciences, and Environmental Sustainability, University of Parma, 43123 Parma, Italy; cristina.dallabona@unipr.it

**Keywords:** epigenetics, prescription opioids, pain

## Abstract

Prescription opioids are used for some chronic pain conditions. However, generally, long-term therapy has unwanted side effects which may trigger addiction, overdose, and eventually cause deaths. Opioid addiction and chronic pain conditions have both been associated with evidence of genetic and epigenetic alterations. Despite intense research interest, many questions about the contribution of epigenetic changes to this typology of addiction vulnerability and development remain unanswered. The aim of this review was to summarize the epigenetic modifications detected in specific tissues or brain areas and associated with opioid prescription and misuse in patients who have initiated prescribed opioid management for chronic non-cancer pain. The review considers the effects of opioid exposure on the epigenome in central and peripheral tissues in animal models and human subjects and highlights the mechanisms in which opioid epigenetics may be involved. This will improve our current understanding, provide the basis for targeted, personalized pain management, and thus balance opioid risks and benefits in managing chronic pain.

## 1. Introduction

Chronic pain represents significant public health concerns and prescription opioids are a common treatment option for cancer pain management, for end-of-life treatment, in relation to surgery, and for short-term use in severe acute/chronic pain conditions not related to cancer [1]. The non-medical use of opioids and their negative health consequences among people who use drugs have been studied since 2007 after the spread of the opioid crisis. However, in the last few years, we have witnessed a new opioid crisis, even among young people and categories of workers, particularly in North America, the Middle East, Asia, and Africa. This crisis is related to the non-medical use of prescription opioids that can result in opioid misuse, defined as “use contrary to the directed or prescribed pattern of use, regardless of the presence or absence of harm or adverse effects” [2]. Signs of an increase in methadone, buprenorphine, fentanyl, codeine, morphine, tramadol, and oxycodone misuse and the increased prescription rates for opioids for pain management have also been observed in Europe resulting in an increase of vulnerable cohorts of long-term opioid users [3]. The central issue is that long-term opioid therapy is associated with many side effects such as addiction, development of tolerance, and opioid-induced hyperalgesia. In addition, in 2018 more than one-third of overdose deaths involved pharmaceutical opioids with the number of overdose deaths rising from 3442 in 1999 to 17,029 in 2017 [4,5].

Opioid drugs act not only in nociceptive processes but also in modulating gastrointestinal, endocrine, and autonomic functions, as well as in affecting cognition and reward systems [6]. The relationship between pain states and substance abuse/misuse has been recently examined. Opioids carried an increased susceptibility to abuse even during initial exposure for pain treatment; in particular, when too many opioid drugs are prescribed for conditions not supposed to be treated by opioids or if healthcare systems are not set up to control the number of opioid prescriptions to an individual patient (doctor shopping) [7,8,9].

Nevertheless, it is important to note that the lack of consistent findings regarding the identification of personal risk factors that may predict opioid misuse in chronic pain patients was recently evidenced in the literature [10]. Among the possible risk factors, the individual genetic variability in conjunction with chronic pain, both affecting stress and reward systems, lead to differential responses to opioids and may determine the transition risk from therapeutic use to opioid addiction. Addiction is a multifactorial condition as both genetics and psychosocial factors can trigger opioid addictive behaviors. Polymorphisms in the *μ*-opioid receptor 1 (*OPRM1*), the cytochrome P450 2D6 (*CYP2D6*), the catechol-O-methyl transferase (*COMT*) genes and in the ATP-binding cassette family genes have been found to be associated with differences in morphine consumption and metabolization process [11]. The main environmental factors important for developing opioid/substance abuse are described as psychiatric medication prescriptions, mood disorders, specific mental health diagnoses, and adverse childhood experiences [12,13].

An intrinsic mechanism that can be induced and modulated by environmental stimuli and that represents the bridging gap between environment and genetics is epigenetics [14]. Specific epigenetic modifications, such as alterations in DNA methylation patterns, non-coding RNAs (ncRNAs) expression, higher levels of permissive histone acetylation, and lower levels of repressive histone methylation, have been widely evidenced and associated with addictive behavioral changes in the brain’s reward circuitry in opioid users. The identified associations allowed to hypothesize causal links [15]. In addition, the epigenetic consequences of widespread synthetic opioid consumption in future generations, the so-called transgenerational effects, have recently been described [16].

The aim of this review was to report the collected data from published studies on the identified epigenetic modifications associated with opioid prescription and misuse in patients who have initiated prescribed opioids for pain.

## 2. Materials and Methods

The literature search strategy included the use of PubMed, MEDLINE, Google Scholar, and Embase databases. Publications up to February 2021 were included. Peer-reviewed articles were collected based on the search terms: prescription opioids, pain, epigenetics, gene expression, DNA methylation, non-coding RNAs, chromatin, and histone modifications. Relevant literature was selected based on headline and abstract. A comprehensive search was conducted in order to provide an outline of the detected epigenetic changes related to the administration or assumption of prescription opioids in the context of pain. We also used broad search terms to increase the probability of precise identification of the target articles (Table 1, Table 2 and Table 3).

All the publications collected in the literature search phase underwent abstract screening to determine eligibility for full-text data extraction. To be eligible for data extraction, studies met the following inclusion criteria: (1) the selected publications were in English language only; (2) the samples were composed of animal models to study chronic pain or human subjects with chronic noncancer pain (persistent pain lasting longer than 3 months), (3) participants were exposed to or were using prescription opioids, (4) the abstract listed one or more of the following terms in reference to potential epigenetic changes: DNA methylation, histone, chromatin, non-coding RNAs, microRNAs, gene or protein expression. Studies related to genetic polymorphisms, which are changes in the DNA sequence, were excluded from the review.

Finally, to identify potentially relevant studies not detected through the main screening, a few articles were selected from other reviews.

A thorough screening of the collected papers was conducted based on the inclusion criteria and quality of the experiments (methods typology to detect the epigenetic changes, adequate description of study methodology, sample size, and inclusion/exclusion criteria). Analyzing the identified papers, three main categories were evidenced and grouped in Table 1, Table 2 and Table 3, respectively: (i) studies investigating epigenetic changes in animal models (16), (ii) studies involving human subjects (3), and (iii) studies related to intergenerational or transgenerational prescribed opioid effects (5). Among the three studies on human subjects, one was related to cancer pain patients [35]. Nevertheless, it was included because considered relevant.

Moreover, within the three subgroups, the articles were listed in the tables by the type of opioids (morphine, oxycodone, etc.). It thus became clear that the identified studies primarily investigated the morphine exposure effect on gene expression and chromatin modifications. Among the studies exploring oxycodone exposure in animal models, experiments measuring DNA methylation and DNA hydroxymethylation were selected. The few studies that considered these effects on human subjects were focused on DNA methylation. Interestingly, the screening revealed a group of articles related to the intergenerational and transgenerational effects of the epigenetic marks identified. Hence, the related remarkable information was described in a dedicated paragraph. In light of the few human studies identified, a paragraph about the epigenetic research related to heroin users was included to represent how these modifications have been studied in human subjects and to give future directions for the prescription opioid research related to different pain conditions.

## 3. Results

### 3.1. Prescription Opioids Pain Relievers

Opioid receptors are widely distributed both centrally and in the periphery, particularly in the periaqueductal grey, *locus ceruleus*, rostral ventral medulla, and in the *substantia gelatinosa* of the dorsal horn. The major mechanism through which opioids relieve pain is the stimulation of descending inhibitory neurons through activation of the μ opioid MOP receptors. Common prescription opioids responsible for this opioid-induced analgesia effect are morphine, codeine, tramadol, fentanyl, hydromorphone, buprenorphine, hydrocodone, oxycodone, oxymorphone, methadone, and tapentadol [41].

Opioid medications are often prescribed for acute episodes of pain for short-term use or for cancer-related pain. Opioids are also used for chronic non-cancer pain in selected cases when non-opioid and adjuvant therapies have failed and other pain medications have proven ineffective. These drugs are defined as highly effective and safe analgesics when used appropriately and included in a multifaceted strategy by competent clinicians [42].

However, since opioid-induced pain relief and addiction do not act through distinct mechanisms in distinct brain areas, opioid drugs could not only have a simple analgesic effect but also affect or compromise the ability to feel pleasure and socialize as well as the reward system [43]. Neural changes were likened between chronic pain and long-term substance abuse; in fact, dysfunctional learning may trigger both these pathological states, producing an extensive reorganization in chronic pain and converting functional rewards into the craving characteristic of addiction [44].

In light of the fact that the opioid analgesic efficacy often decreases with continuous use and that patients with refractory complex chronic pain are at high risk of abuse, a two-level strategy might be set up to contrast the opioid crisis. The first level would include prescription monitoring programs and dose limitations to prevent abuse/misuse; the second could increase research at the molecular level to identify the central and peripheral mechanisms underlying the drug action and to explore precision medicine options.

### 3.2. Epigenetics and Prescription Opioids

The term epigenetics refers to the study of heritable changes in the gene function that do not involve changes in the DNA sequence [45]. Epigenetics includes three main mechanisms: DNA methylation and chromatin-related modifications, both affecting the ability of transcriptional machinery to access the DNA tightly packed into chromatin, and non-coding RNAs (ncRNAs).

DNA methylation, the addition of a methyl group on the 5th carbon of the DNA cytosine, is shown particularly relevant in CpG islands, regions of the genome containing a large number of CpG dinucleotide repeats [46]. It regulates gene expression facilitating the recruiting of proteins involved in the gene repression or inhibiting the binding of transcription factors to DNA [47]. Thus, DNA methylation plays a critical role in the regulation of gene expression. Whole-genome methylation profiling has made it possible to better explore demethylation and de novo methylation in the maternal and paternal genomes during development. This enables highlighting of more complex dynamics within the heterogeneous methylation level at CpG-rich promoters in different cell types. Many unannotated sequences and inactive transposons affected by this epigenetic mark were revealed [48]. Opioids have been demonstrated to stimulate DNA methylation. One study identified the global DNA methylation at LINE-1 as significantly correlated with increased chronic pain. Thus, it was hypothesized that opioid analgesics might be causally associated with increased genome-wide DNA methylation [49].

The chromatin modifications include those related to histone tails, such as histone methylation, chromatin remodeling, and post-translational modifications affecting electrostatic nucleosome interactions. These changes have been clinically and pre-clinically evidenced to be associated with opioid exposures [15]. Concerning prescription opioids, oxycodone exposure was shown to induce long-term epigenetic consequences in the ventral tegmental area (VTA) of the developing brain with an enrichment of the repressive histone mark, H3K27me3, in prolonged oxycodone withdrawal and with consistent inhibition of the gene regulation [28]. The other chromatin modifications are related to the chromatin structure that is hierarchically folded at different levels in the nucleus with a 3D organization [50]. Previous works evidenced a correlation between the effects of opioids and chromatin remodelers such as CREB, Sox10, and BRG1 [51,52,53]. However, no studies have thoroughly explored prescription opioids’ effect on chromatin structure remodeling.

The third important regulator of transcriptional activity is represented by ncRNAs [54], i.e., RNA not translated into proteins. ncRNAs act through a variety of mechanisms such as post-transcriptional silencers or activators. Moreover, they are involved in regulating protein-coding and non-coding genes’ expression, in the guide of chromatin-modifying complexes to specific genomic loci, in the modulation of transcriptional programs, and providing molecular scaffolds [55]. ncRNAs have already been correlated with opioid exposure. In particular, morphine, fentanyl, and heroin were found to modulate the expression of specific micro-RNAs [25,56]. Additional studies are required to understand the functional consequences of these epigenetic changes.

The nociceptive response was demonstrated to activate these epigenetic mechanisms by modulating pain genes and possibly mediating the transition from acute to chronic pain. Studies highlight that also opioids are involved in diverse types of epigenetic regulation and thus they might influence the analgesic effects or the increased risk of continued opioid intake and development of a substance use disorder following long-term opioid therapy [57,58].

However, the identification of specific factors associated with the individual opioid response or with side effect vulnerability has just been launched. The molecular mechanisms through which some individuals develop negative consequences associated with prolonged prescription opioid use, including hyperalgesia, addiction, sleep problems, hypogonadism, fractures, and surgical failures [59,60] are poorly understood. The following paragraphs provide an overview of the animal and human studies investigating epigenetic changes associated with opioid therapy initiated for pain relief. The studies are illustrated in Table 1; Table 2, respectively.

#### 3.2.1. Animal Models’ Studies

The studies exploring the effect of painkillers in animals mainly investigated gene and protein expression changes following morphine exposure. One of the first studies determined the analgesic tolerance to morphine in mice with congenic genotypes for a proximal region of chromosome 10 that includes the *OPRM1* gene. The *OPRM1* is responsible for a significant portion of the variance in the response to morphine. Pharmacologically relevant doses of morphine were chronically administered. Microarray gene expression profiling of brain areas, relevant for nociception, analgesia, conditioned reinforcement, and drug abuse (prefrontal cortex, ventral striatum, temporal lobe, and periaqueductal gray), revealed specific genes (*B4GALT1*, *HBA2*, *TTK*, *H19*) and gene networks associated with analgesic tolerance and predisposition. Instead, the dopamine D3 and D4 receptors, the dopamine transporter, *DARPP-32*, and calcyon genes were found to be differentially regulated by morphine, and it has been hypothesized that they are associated with morphine-rewarding effects. All these genes modified by chronic morphine administration play a role in the neuroadaptation pathways [17]. The effect of repeated morphine administration was also shown to reduce the expression of the histone methyltransferase G9a, which catalyzes the dimethylation of histone H3 at lysine 9 (H3K9me2). Decreased H3K9me2 in mouse *nucleus accumbens* (NAc) was also reported at both the global level and in specific loci. In particular, reduced H3K9me2 was revealed in the *FosB* gene with subsequently enhanced activity of the encoded protein ΔFosB, a key player in drug behavioral response, and in the glutamatergic genes, such as *GRIN2A, GRM5, GRM8*, with corresponding increases in their mRNA expression. The study also demonstrated that downregulation of G9a after chronic morphine enhanced locomotor sensitization and sensitivity to the rewarding effect of the drug. At the same time, G9a downregulation reduced the physical dependence and delayed the morphine analgesic tolerance development, highlighting its crucial role in maintaining normal gene transcription [18].

Other mechanisms were also proposed to be related to morphine reward and tolerance. Concerning morphine-seeking behavior and reward, a mechanism that may contribute to increased response in the behavioral preference for opioids under pain conditions was proposed. In fact, persistent pain and repeated morphine upregulated MeCP2, a transcriptional regulator which binds to methylated CpG sites of DNA, in the mouse central nucleus of the amygdala (CeA) [19]. This structure, belonging to the limbic system and accounting for pain-related emotional responses, is thus called the nociceptive amygdala [61]. In mice with persistent pain or morphine-induced CPP (conditioned place preference), MeCP2 was suggested to enhance the brain-derived neurotrophic factor (*BDNF*) expression through the repression of G9a and a subsequent reduction of the repressive mark H3K9me2. Further, knockdown of G9a evidenced an increased sensitivity to pain and to morphine reward-related behavior [19].

Subsequent experiments of morphine self-administration in rats with persistent inflammatory pain demonstrated that after morphine withdrawal GluA1 subunits of glutamate AMPA receptors, known to be upregulated in pain conditions with the potentiated nociceptive transmission, were overexpressed in CeA. The proposed mechanism by which pain determines GluA1 upregulation was the decreased recruitment of MeCP2 onto *GRIA1* promoters (the GluA1 gene) with the subsequent de-repression of *GRIA1* expression and enhancement of the morphine-seeking behavior in rats with pain experience [20].

Concerning analgesia, some studies reported epigenetic changes associated with neuropathic pain and morphine analgesia. First, it was demonstrated that the diminished expression of the μ-opioid receptors (MOR), observed following nerve injury, was mediated by an epigenetic mechanism. In fact, nerve injury consistently increased the G9a action and thus the levels of H3K9me2 in the promoter of the *OPRM1* gene in the dorsal root ganglia (DRG); this resulted in condensed chromatin and gene silencing and a diminished opioid analgesic effect. Consistently, G9a inhibition in the injured DRG rescued the MOR expression and restored the morphine effect on pain hypersensitivity induced by nerve injury [21]. Li and coworkers (2017) identified specific genes in which the modulating expression allows improvement of morphine analgesia after chronic constriction injury (CCI), which is a rat model causing persistent pain hypersensitivity after peripheral nerve injury to mimic trauma-induced neuropathic pain [22]. Specifically, CCI and thus neuropathic pain increased mRNA and the protein levels of *C/EBPβ* (CCAAT/enhancer-binding protein β), a transcription factor regulating gene expression in immune and inflammatory responses [62]. *C/EBPβ* was shown to indirectly inhibit MOR and the voltage-gated K+ channel Kv1.2 abundance in the ipsilateral DRG by activating the G9a gene (*EHMT2*), thus resulting in condensed chromatin and gene silencing. To confirm that *C/EBPβ* contributes to neuropathic pain through epigenetic silencing of Kv1.2 and MOR, it was demonstrated that blocking *C/EBPβ* the morphine analgesia following CCI was rescued [22].

Another fundamental aspect explored was the long-term neural and behavioral consequences after opioid exposures in the context of histone tail modifications. The repressive histone tail mark H3K27me3 was identified specifically in adolescent mice VTA following oxycodone exposure with consistent inhibition of gene regulation indicating an age-dependent oxycodone effect [28]. Instead, morphine exposure revealed that histone deacetylase (HDAC) inhibition in the basolateral amygdala (BLA) plays a crucial role in opioid-associated memories. In particular, the administration of an HDAC inhibitor in rats facilitated acquisition and expression of morphine-induced CPP and was associated with a general increase in histone H3 lysine14 (H3K14) acetylation with an upregulation of the *BDNF*, *FosB* gene expression and with CREB (cAMP-responsive element-binding protein) activation; all factors implicated in drug addiction and learning memory [23].

Moreover, the *BDNF* has also been identified among DNA methylation-regulated genes. Barrow and coworkers (2017) observed that acute morphine exposure induced significantly decreased methylation in *BDNF* and *IL1B*, and significantly increased methylation in *IL6* and *NR3C1*. While chronic morphine exposure induced significantly decreased methylation of *BDNF* and *COMT* and significantly increased methylation of *IL1B* and *NR3C1* in different rat brain regions. The study also analyzed global DNA 5-hydroxymethylation observing a significant increase in the cerebral cortex, hippocampus, and hypothalamus, as well as a significant decrease in the midbrain [24].

It is also important to report the role that micro-RNAs (miRNAs) might play in opioid response and pain. In fact, it has been observed that commonly used analgesics altered their expression [63]. One example is miR-339-3p, the expression of which was consistently increased by both morphine and fentanyl. Then, it was demonstrated that miR-339-3p destabilizes MOR mRNA and thus inhibits the MOR protein biosynthesis [25]. In nociception, the altered expression of many miRNAs was described in the context of surgical pain models, and some of them were related to morphine tolerance [63]. Chronic morphine treatment was observed to increase *BDNF* transcription and translation. One study demonstrated that this process, which contributes to the development of chronic tolerance to morphine analgesia in mouse DRG, was mediated by miR-219 by targeting CaMKIIγ [26]. Another study showed intervention of the miR-375 and JAK2/STAT3 pathways in the same process [27].

Other studies focused on the rewarding effects of the widely abused prescription drug oxycodone, exploring related gene expression and DNA methylation changes after a period of chronic oxycodone self-administration. In particular, RNA-sequencing in the NAc and caudate-putamen (CPu) of mice following extended 14-day oxycodone self-administration revealed differential expression of the axon guidance molecule integrins, semaphorins, and ephrins, which may correspond to alterations in the axon-target connections and synaptogenesis and thus to the oxycodone-induced neuroadaptations [29]. In addition, levels of numerous glial-specific and immune cell-specific genes were also found to be altered in the CPu and NAc consistent with a previous study identifying altered expression of genes related to the inflammation and immune functions [30]. Furthermore, two interesting studies in the rat VTA and hippocampus, respectively, demonstrated that the oxycodone induction of CPP acquisition was associated with changes in gene expression and DNA methylation. Specifically, a down-regulation of DNMT1 and up-regulation of TET1-3 were observed in the VTA leading to a decrease in global DNA methylation levels and differential demethylation of the *SYN* and *PSD95* genes with consistent higher expression of these synaptic proteins and the synaptic density [32]. In the hippocampus, global hypomethylation, higher synaptic density, and increased expression of specific synaptic genes, *SYNAPSIN, SHANK2*, and *GAP4*, were observed, too [31]. In addition, these studies suggested the therapeutic potential of oxytocin to prevent oxycodone addiction. In fact, oxytocin pretreatment markedly inhibited the acquisition of oxycodone CPP and restrained stress-induced reinstatement of oxycodone. Consistently, the synaptic density and the global DNA hypomethylation induced by oxycodone were normalized and the transcription of synaptic genes and DNA methylation enzyme genes was restored [31,32].

**Figure 1:**
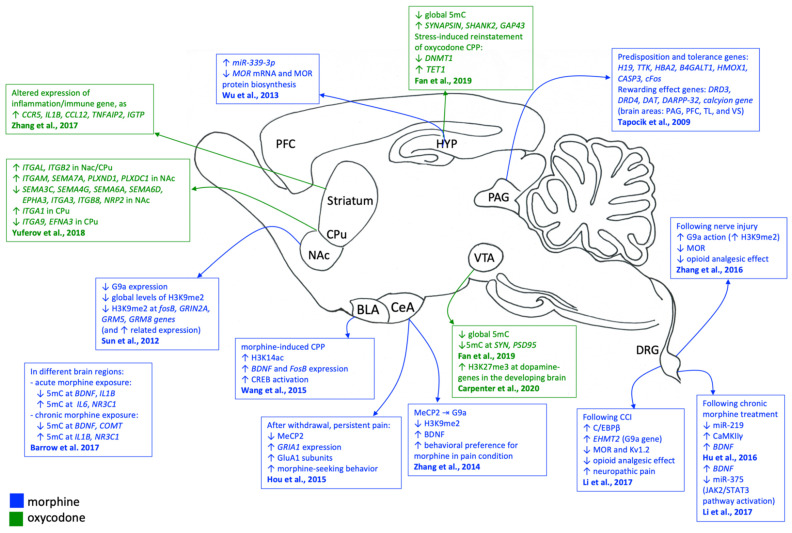
Epigenetic changes detected following chronic prescription opioids exposure in experimental models. Blue color is related to morphine exposure; green color is related to oxycodone exposure. Symbols meaning: ⇥ repression; ↓ decreased expression/repression; ↑ increased expression/activation.

The reported epigenetic changes in connection with the related brain areas are reported in Figure 1.

#### 3.2.2. Human Studies

Research on epigenetic effects of prescription opioids in humans is still in its infancy and mainly focuses on DNA methylation changes in peripheral tissues. The reason for the scarce results may be seen in light of the difficulties to measure epigenetic modifications in the human brain: the majority of the differentially methylated patterns was detected in peripheral tissues that may not be indicative of alterations in reward and pain pathways. However, other studies identified overlapping epigenetic signatures in peripheral and central tissues indicating that blood cells could represent a source of non-invasive biomarkers for opioid misuse susceptibility or prediction also in chronic pain patients [64,65]. The studies described in this section are reported in Table 2.

Three differentially methylated CpG sites in genes implicated in chromatin remodeling, DNA binding, cell survival, and cell projection were identified with decreased DNA methylation associated with opioid dependence development. Total DNA derived from whole blood samples was compared between opioid-exposed subjects developing dependence and not developing dependence. In particular, the top CpG sites identified were: cg17426237 related to *PARG* (the poly ADP-Ribose glycohydrolase gene), which is highly expressed in the human brain; cg04983519 at the *EHMT2* gene encoding for the histone methyltransferase G9A involved in transcriptional repression; cg21381136 at the *RERE* gene (arginine-glutamic acid dipeptide repeats gene) that was shown to interact with *EHMT2*; and cg18177613 related to *CFAP77* (the Cilia And Flagella Associated Protein 77 gene) [33]. Other CpGs were evidenced in genes implicated in the metabolism (*OSBPL9*, oxysterol binding protein-like 9) and the protein binding/axon guidance (*TUBA1C*, tubulin α 1c).

Interestingly, one study explored the early epigenetic effect of therapeutic opioids at three time points; one before and two after opioid self-administration in 33 subjects undergoing standard dental surgery and never exposed to opioids. The study demonstrated how the response to opioids occurred within the first few days to weeks following drug exposure as dose-dependent hypermethylation was observed in nine CpGs analyzed in the *OPRM1* promoter. At a genome-wide level, the DNA methylation changed in 1701 CpGs belonging to genes related to natural killer cell-mediated cytotoxicity and cellular defense response; in particular, the methylation decreased within a week (2.7 ± 1.5 days) of the last opioid dose and re-increased 39 ± 10 days after the last opioid dose in the majority of these CpGs (>65%), while the other CpGs had an opposite trend [34].

Even if related to cancer pain, the experiment of Kringel and coworkers (2019) should also be considered because of the prospective design, which may provide more definitive answers to questions of causality. The study confirmed the predictive utility of epigenetic testing in a sample of 140 women who had undergone breast cancer surgery [35]. DNA methylation quantification was performed in the retrotransposon LINE1, a retrotransposon used as a marker of global DNA methylation being in approximately half a million copies across the human genome [66]. No association between the DNA methylation and persistent postoperative pain was evidenced when explored in two key players of glial/opioid intersection: the toll-like receptor 4 (*TLR4)* and the μ-opioid receptor *(OPRM1*). However, the global level of DNA methylation was shown to be predictive for persistent pain, comparing 70 women with persistent pain diagnosis with 70 matched controls who had not developed persistent postsurgical pain [35].

### 3.3. Intergenerational and Transgenerational Epigenetic Effect of Prescription Opioids

Evidence suggests that epigenetic changes might be transferred to the next generations. Furthermore, germ cells express opioid receptors strengthening the potential of this research. Experiments related to the reactivity to opioids suggest that alterations in the endogenous opioid system can persist in the progeny of parents exposed to opioids and in the following generations [16]. Most of the research carried out in this field involved animals.

Both intergenerational and transgenerational epigenetic inheritance refers to the transmission of non-DNA base sequence information between generations; but only in the second case, the effects are found in generations that were not exposed to the initial signal or environment that triggered the change [67,68]. Intergenerational evidence already shows that having at least one morphine-treated parent was associated with reduced avoidance memory, visceral, acute, and persistent nociception, and anxiety-like behavior. Anxiety-like behavior increased in female offspring with both parents having been morphine-treated, suggesting an epigenetic role of maternal inheritance [69]. Indeed, prenatal oxycodone exposure was shown to alter the gene expression in neonatal rats, in particular in the endothelin receptors genes involved in the central nervous system (CNS) development [36]. One research, involving human participants, considered in utero opioid (methadone or buprenorphine) exposed infants: an increased methylation within the *OPRM1* promoter was revealed. This change was found to be associated with a worse outcome of neonatal abstinence syndrome [37]. A more recent study conducted RNA sequencing of brain-derived extracellular vesicles, which are miRNA cargo [70], and the role of these vesicles as pain biomarkers was explored. The authors identified miRNA signatures associated with brain development in offspring exposed to oxycodone *in utero*; interestingly, the detected miR-504 increase correlated with increased expression of *DRD1* and *DRD2* receptors in synapses. [38]. Gilardi and coworkers (2018) have recently summarized the transgenerational effects of parental exposure to synthetic opioids on the subsequent generations. Interestingly, two studies explored the inheritance of gene expression changes through generations following morphine exposure [39,40]. One experiment evaluating F1 and F2 progeny without direct fetal exposure demonstrated that adolescent opiate exposure could affect the vulnerability of future offspring. The authors found an attenuated locomotor sensitization in response to repeated dopamine D2 receptor (D2R) activation mediated by the administration of the D2R activator quinpirole with coupled upregulated kappa opioid receptor and D2R gene expression within the NAc in both F1 and F2 progeny. This indicates a significant effect in the progeny of females exposed to opiates during adolescence [39]. Another experiment tested if maternal exposure to morphine prior to pregnancy-induced alterations in drug abuse in the subsequent generations. Genes related to synaptic plasticity and myelin basic protein were found differentially expressed and some changes persisted into the subsequent (F2) generation [40].

The studies investigating intergenerational and transgenerational epigenetic changes following prescription opioid exposure are reported in Table 3.

### 3.4. What Can We Learn from Heroin Epigenetics?

Even if the research on how epigenetic modifications could contribute to addiction development has been less comprehensive for heroin compared with psychostimulants [15], important findings have been revealed. As a reflection of the excessive dopamine neurotransmission in the NAc, the expression of genes involved in processes related to neurotransmitter release, including the synaptotagmin 1, *SNAP25*, synapsin 2a, and 2b, pallidin, amysin, and the endogenous opioid peptide prodynorphin genes, were found significantly reduced in heroin abusers [71]. Moreover, higher DNA-methylated regions were identified in the PFC of heroin users, specifically axonogenesis- and synaptic-related gene of glutamatergic neurons, whereas hypomethylation was detected in transcription factor and expression regulation genes [72]. Also in rat models, a hypomethylation and an associated transcriptional upregulation in the γ-aminobutyric acid type A receptor subunit delta (*GABRD*) gene were found in the NAc following heroin self-administration. The study proposed new therapeutic strategies based on manipulation of the enzymes responsible for this epigenetic modification, such as DNMT1 and DNMT3A [73]. Pharmacological manipulation was also suggested by Egervari and coworkers. The research performing a genome-wide assessment of chromatin accessibility in the human striatum of heroin users revealed that heroin-induced expression of the tyrosine kinase *FYN* gene, its kinase activity, and the phosphorylation of its target. Interestingly, inhibition of FYN activity attenuates heroin self-administration in rats [74].

Novel therapeutic targets were also proposed among histone tail modifications [75,76]. For example, H3K9ac, which is associated with transcriptional activation, was found significantly elevated in the Brahma/SWI2-related gene-1 (*BRG1*) with related higher expression of this gene in the mPFC after heroin self-administration [77]. The activation of histone acetylation has already been proposed as a pharmacological strategy for treating heroin-seeking behavior. The levels of histone H3 acetylation at lysine (H3K18) and H4 acetylation at lysine 5 or lysine 8 (H4K5 or H4K8) in the NAc were remarkably increased during the reinstatement and were strengthened after intracerebroventricular injection of an HDAC inhibitor [78].

The role of miRNAs, 18-25 nucleotide non-coding sequences with a wide range of regulatory roles in gene expression and neuronal functions, was also investigated in heroin-seeking behavior. MiR-218 was found to regulate many neuroplasticity-related genes and target the methyl CpG-binding protein 2 (Mecp2), thus inhibiting heroin-induced reinforcement [56].

Heroin studies also recall the role of environmental factors affecting the vulnerability to the drugs of abuse response and may also modulate the neuro-anatomical/neuro-chemical impacts of uncontrolled drug use and relapse propensity. Environmental enrichment, such as the presence of alternative rewards that reduce stress and anxiety, was demonstrated to significantly reduce motivational measures of drug-seeking and drug-taking behavior, also in heroin self-administration models. From a molecular point of view, it was demonstrated that both forms of reward, heroin or environmental enrichment, reduced the *EGR1* and *EGR2* expression and increased the methylation of the *EGR2* gene promoter. *EGR1* and *EGR2* are two early response genes sensitive to heavy substance use and the environment [79].

Even if the heroin epigenetics research seems to be at an advanced stage compared with the epigenetic research of prescription opioids, it should be noted that many questions remain unanswered. The length of time to observe the epigenetic changes induced by chronic heroin, especially in the peripheral tissues that are more suitable for biomarker identification, still needs further evaluation [80].

## 4. Discussion

**Figure 2:**
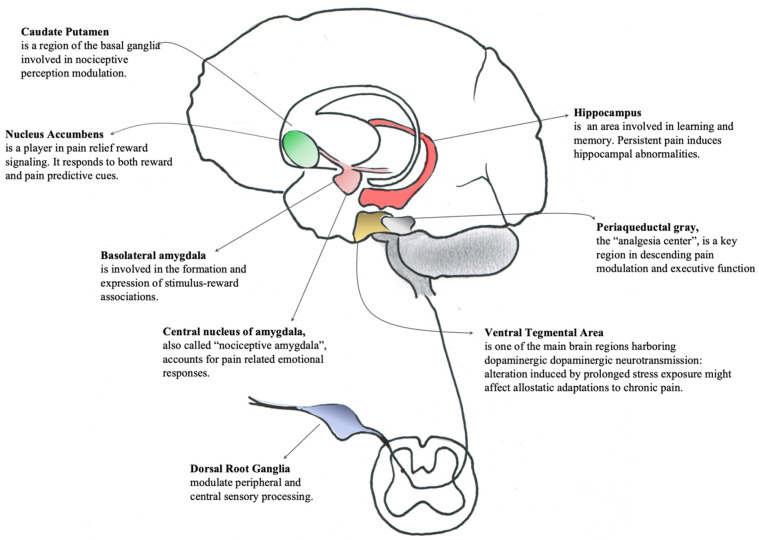
Evidenced areas involved in prescription opioids action and their role in pain perception.

The present overview elucidates the detected epigenetic modifications following prescription opioids for pain. It is worth noting that the majority of studies focused on morphine administration in animal models. Few studies analyzed epigenetic changes in humans after different opioid administration, however, the evidenced brain areas involved in prescription opioids action and their role in pain perception are reported in Figure 2.

One of the most interesting results is the role of the histone methyltransferase G9a, which specifically methylates the Lysine 9 of histone H3 (H3K9me1 and H3K9me2). G9a seems to act as a crucial player balancing gene expression and resulted in turn differentially regulated by morphine exposure or pain. In fact, repeated morphine administrations downregulate the *G9a* expression with subsequent gene expression activation of ΔFosB and glutamatergic genes [18]. Interestingly, in pain conditions, the upregulated expression of the GluA1 gene was traced back to reduced recruitment of the methyl CpG binding protein 2, MeCP2 [20]. GluA1 gene encodes the AMPA receptor subunit glutamate A1 modulating the trafficking and integration of AMPA receptors within synaptic membranes [81]. Consistently, the methyl CpG-binding protein 2 MeCP2 regulates the G9a activity both in morphine administration and pain conditions [19].

By contrast, nerve injury models and CCI resulted in enhanced *G9a* expression and subsequent *OPRM1* gene silencing in DRG [21,23] and the process could be mediated by the transcription factor C/EBPβ acting in immune and inflammatory responses [23].

The role of G9a was also evidenced in peripheral samples of opioid-dependent subjects in which a decreased DNA methylation was evidenced in a specific CpG of the gene. Even if DNA methylation is mainly associated with gene repression, it is difficult to predict the consequence of a single CpG methylation on the gene and protein expression and this result should draw further attention to the role of G9a.

This evidence corroborates the hypothesis that epigenetically mediated G9a regulation is a common adaptive response of neurons to compensate for the negative effects of chronic drug use on the brain [82], and this might be true also for the negative effects of pain.

Another key element detected from the literature analyzed is BDNF and its miRNA-mediated regulation. Chronic morphine administration induced an increased *BDNF* expression with associated higher global 5hmC and the process was shown to involve specific miRNAs. Moreover, BDNF is a well-known mediator of the mechanism through which neuronal plasticity is affected by environmental experiences [83] and should be considered in association with the environment, which has mainly been explored in heroin-related research. Environmental risk factors for opioid misuse, abuse, or addiction have been identified as psychiatric comorbidities, substance abuse, adverse childhood experiences, general social or family environments, and psychosocial vulnerability [84,85]. Interestingly, pain and pain-related brain changes are also highly affected and can be reduced by socially and physically enriched environments, exercise, and mood [86]. In this context, it is important to recall the specific interactions evidenced between the nervous and the immune systems following prolonged opiate administration: the opiates-induced change in the expression and distribution of opioid receptors in the brain [87] are accompanied by the generation of autoantibodies to opiate and AMPA receptors [88,89]. This immune adaptive response has been shown to affect pain threshold, to stimulate a compulsive behavior in animal models, and thus might reveal biomarkers of the neurobehavioral consequences of repeated drug exposure [90].

These findings highlight the complex interplay between pain, the adaptive responses of the immune system, addiction, and the environment, offering a possibility to reduce or prevent the negative consequences of chronic pain and prescription opioid abuse by environmental and lifestyle factors.

It is well known that the environment (e.g., diet, exposure to chemicals or toxins, psychological stress, etc.) can influence the epigenome and gene expression. In contrast, it is still a controversial and debated issue whether the environment experienced during our lifetime may be inherited intergenerationally or transgenerationally and thus if it can have an impact on the health of the descendants. However, new insights are emerging [91,92,93,94]. The impact of opioid use on the offspring has been investigated and accumulating evidence suggests that alterations in the endogenous opioid system can persist in the progeny of parents exposed to opioids and in the following generations [52] making it even more important to carefully consider the epigenetic effects of prescription opioid abuse.

Although few, the human studies reveal crucial aspects to be considered for future research design. In fact, the cited experiments involved the comparison of subgroups of opioid exposed subjects, who developed or not developed the diseases (persistent pain or opioid dependence). In other cases, the epigenetic analyses were conducted at different time points, before and after opioids assumption. These strategies allow to prove the strength of an association between a disease and potential causal factors: prospective longitudinal investigations are encouraged in prescription opioids and pain research to identify epigenetic biomarkers.

The studies on heroin point toward the possibility of developing new epigenetic pharmacological approaches. In particular, research should fully explore the role of DNMT, HDAC, and other potential therapeutic targets identified in the context of heroin rewarding and heroin-seeking behavior in order to determine if these targets might be considered in the research of prescription opioid use disorders. It should be noted that the findings of the studies oriented towards identifying promising therapeutic targets for heroin use disorders mainly evidenced the GABAergic and glutamatergic transmission [95]. Importantly, these studies point towards the importance of considering the epigenetic signatures as a whole correlating gene expression to the epigenetic marks which might modify the gene transcription including DNA methylation, miRNAs, and histone changes as well as chromatin accessibility.

## 5. Conclusions

Our review summarizes the epigenetic factors that are associated with prescription opioids used for pain. Specific biomarkers that could serve as potential therapeutic targets or factors allowing to reveal the complex molecular mechanisms underlying prescription opioid addiction and pain are reported. Of special interest is the histone methyltransferase G9a, the role of which should be explored in detail. The studies conducted in humans are insufficient for the purpose of biomarker identification and the underlying epigenetics remains poorly understood. From these studies, the first evidence of the causal relationship between therapeutic opioid administration and epigenetic changes was revealed and research should further explore the temporal dynamics of these modifications in response to both prescription opioids administration and pain.

Exploring these pathways could reveal the molecular mechanisms and potential therapeutic targets for preventing chronic pain and addiction.

## Figures and Tables

**Table 1:** Epigenetic changes after prescribed opioid exposure in experimental models.

Opioids	Tissues/Sample	Epigenetic Methods	Change	Animals	Findings	PMID	Authors
Morphine	Brain tissues (PAG, PFC, temporal lobe, and ventral striatum)	Microarray gene expression profiling and pattern matching	Gene expression	Adult male mice	The development of tolerance is influenced by a region in *OPRM1* gene. The genes epigenetically modified by chronic morphine administration are mainly related to neuroadaptation.	19386926	Tapocik et al., 2009 [17]
Morphine	NAc	Chromatin immunoprecipitation followed by massive parallel sequencing	H3K9me2 distribution in NAc in the absence and presence of chronic morphine	9 to 11-week-old C57BL/6J male mice or G9afl/fl mice	Chronic morphine decreases G9a expression and H3K9me2 at global level and in specific loci in mouse NAc.	23197736	Sun et al., 2012 [18]
Morphine	Central nucleus of amygdala	Chromatin immunoprecipitation	Gene and protein expression	Female mice with persistent and acute pain	Persistent pain and repeated morphine upregulate the transcriptional regulator MeCP2. MeCP2 enhances *BDNF* expression and represses G9a action and its repressive mark H3K9me2 in CeA.	24990928	Zhang et al., 2014 [19]
Morphine	Central nucleus of amygdala	Chromatin immunoprecipitation	Gene expression	Rat model of morphine self-administration	The repression of GluA1 function by MeCp2 is proposed as a mechanism for morphine-seeking behavior in pain experience.	25716866	Hou et al., 2015 [20]
Morphine	Dorsal root ganglia and spinal cord tissues	Quantitative RT-PCR, Western Immunoblotting and ChIP-PCR	Gene and protein expression, histone modifications analysis	Male Sprague-Dawley rats SNL (spinal nerve ligation) model	G9a contributes to transcriptional repression of MORs in primary sensory neurons in neuropathic pain. G9a inhibitors: potential treatment of chronic neuropathic pain	26917724	Zhang et al., 2016 [21]
Morphine	Dorsal root ganglia	Quantitative RT-PCR and Western Blot	Gene and protein expression	Adult male CD-1 mice	Neuropathic pain increases C/EBPβ expression. C/EPBβ activates the G9a gene, that epigenetically silences Kv1.2 and MOR genes. Blocking the induced increase in C/EBPβ in the DRG, morphine analgesia after CCI is improved.	28698219	Li et al., 2017 [22]
Morphine	Basolateral amygdala	Quantitative RT-PCR and Western Blot	Gene and protein expression	Male Sprague–Dawley	Increase in H3K14ac together with upregulation of the *BDNF* and *FosB*; and CREB activation.	24829091	Wang et al., 2015 [23]
Morphine	Rat brain regions	Pyrosequencing	DNA methylation (5mC) and global DNA 5-hydroxymethylation (5hmC)	Male Wistar rats	Acute and chronic exposure is associated with significantly decreased/increased 5mC at specific genes (*BDNF, IL1B, IL6, NR3C1, COMT*). Global 5hmC levels increase in the cerebral cortex, hippocampus, and hypothalamus, but decrease in the midbrain.	29111854	Barrow et al., 2017 [24]
Morphine, phentayl	Hippocampus	RNAseq	Gene and protein expression	Mice chronically treated with μ-opioid agonists	The increased expression of MiR-339-3p inhibits intracellular MOR biosynthesis and acts as a negative feedback modulator of MOR signals.	23085997	Wu et al., 2013 [25]
Morphine	Dorsal root ganglia	Quantitative RT-PCR and Western Blot	Gene and protein expression	Male CD-1 mice treated with morphine to establish systemic chronic tolerance to morphine anti-nociception	MiR-219 contributes to the development of chronic tolerance to morphine analgesia by targeting CaMKIIγ and enhancing CaMKIIγ-dependent brain-derived neurotrophic factor expression.	27599867	Hu et al., 2016 [26]
Morphine	Dorsal root ganglia	Quantitative RT-PCR and Western Blot	Gene and protein expression	Male CD-1 mice injected with morphine to elicit morphine tolerance	The increased *BDNF* expression is regulated by the miR-375 and JAK2/STAT3 pathway. Inhibition of this pathway decreases BDNF production, and thus, attenuated morphine tolerance.	28603428	Li et al., 2017 [27]
Oxycodone	Ventral tegmental area of the developing brain	Quantitative RT-PCR and chromatin immunoprecipitation	Gene expression and histone modifications analysis	Male offspring of C57Bl/6NTac mice	Adolescent oxycodone exposure increases the repressive mark H3K27me3, at key dopamine-related genes.	33325096	Carpenter et al., 2020 [28]
Oxycodone	Striatum (NAc and CPu)	RNAseq	Gene expression	Mice following extended 14-day oxycodone self-administration	Alterations in the expression of heterodimer receptor, integrins, semaphorins, semaphorin receptors, plexins, selective axon guidance genes.	29946272	Yuferov et al., 2018 [29]
Oxycodone	Dorsal striatum and ventral striatum	RNAseq	Gene expression	Adult male C57BL/6J mice underwent a 14-day oxycodone self-administration	Inflammation/immune genes have altered expression during chronic self-administration of oxycodone	28653080	Zhang et al., 2017 [30]
Oxycodone	Hippocampus	DNA ELISA Kit for total 5mC; quantitative RT-PCR	Global 5mC levels and gene expression	Male Sprague-Dawley rats	The global DNA hypomethylation induced by oxycodone can be reversed through oxytocin and could significantly attenuate the oxycodone rewarding effects.	31526808	Fan et al., 2019 [31]
Oxycodone	Ventral tegmental area	DNA ELISA Kit for total 5mC and OneStep qMethyl™ kit for gene-specific 5mC, quantitative RT-PCR, Western blotting	Global and specific 5mC levels and gene expression	Sprague-Dawley rats	Down-regulation of DNMT1 and up-regulation of TET1-3 lead to a decrease in global 5mC levels and differential demethylation at exon 1 of *SYN* and exon 2 of *PSD95*.	31735530	Fan et al., 2019 [32]

**Table 2:** Epigenetic changes after prescribed opioid exposure in humans.

Opioids	Tissues	Epigenetic Methods	Change	Sample	Findings	PMID	Authors
Opioids	Whole blood	Bisulfite modification and Array-based genome-wide DNA methylation assay	DNA methylation at specific CpG sites	140 opioid dependence cases and 80 opioid-exposed controls	Three genome-wide significant differentially methylated CpGs map to genes involved in chromatin remodeling, DNA binding, cell survival, and cell projection (*PARG*, *RERE*, and *CFAP77* genes).	31801960	Montalvo-Ortiz et al., 2019 [33]
Opioid medication self-administration (hydrocodone, oxycodone, and codeine: 5–30 mg)	Saliva collected at 3 time points	Genome-wide DNA methylation assay and candidate approach	DNA methylation at *OPRM1* gene promoter	33 opioid-naïve participants who underwent standard dental surgery	Hypermethylation of the *OPRM1* promoter is measured in response to opioid use, and such epigenetic restructuring can be induced even by short-term use of therapeutic opioids.	32493461	Sandoval-Sierra et al., 2020 [34]
Remifentanil, oxycodone, codeine	Whole blood	Pyrosequencing at specific CpG sites and LINE1 (global genome-wide DNA methylation assay)	DNA methylation	140 women with persistent pain after breast cancer surgery	The global DNA methylation is shown to be a pain predictive biomarker, providing useful information to allocate the patients to either a “persistent pain” or “non-persistent pain” phenotype.	31775878	Kringel et al., 2019 [35]

**Table 3:** Intergenerational/transgenerational prescribed opioid effects.

Opioids	Tissues/Sample	Epigenetic Methods	Change	Organism	Findings	PMID	Authors
Oxycodone	Rat brains	Quantitative RT-PCR	Gene expression	Timed pregnant Sprague-Dawley rats	Exposed rat pups have lower birth weight and postnatal weight gain and greater congenital malformations. Endothelin B receptor expression is altered in the brain of oxycodone-treated rat pups indicating a possible delay in CNS(central nervous system) development.	26676852	Devarapalli et al., 2016 [36]
Methadone or buprenorphine	Cord blood or saliva	Sequencing of bisulfite-treated DNA	Determination of Percent DNA Methylation	86 infants with Neonatal Abstinence Syndrome from mothers receiving methadone or buprenorphine during pregnancy	High methylation of three specific CpG sites of the *OPRM1* promoter identified is associated with a worse outcome for Neonatal Abstinence Syndrome.	24996986	Wachman et al., 2014 [37]
Oxycodone	Brain-derived extracellular vesicle	RNA sequencing	MicroRNA expression	Male and female Sprague Dawley rats	Distinct miRNA signatures are identified in brain-derived extracellular vesicle at a key stage of brain development in offspring that were in utero and postnatal oxycodone-exposed.	31861723	Shahjin et al. 2019 [38]
Morphine	NAc	Quantitative RT-PCR	Gene expression	Female Sprague-Dawley rats	Increased expression of KOR (K opioid receptor gene) and DRD2 (Dopamine 2 receptor gene) in response to repeated administration of quinpirole (a D2/D3 receptors agonist) in the progeny of females exposed to opiates during adolescence (also observed in the F2 generations).	23314440	Byrnes et al., 2013 [39]
Morphine self-administration	NAc	RNA deep sequencing and qPCR	Gene expression	Female adolescent Sprague Dawley rats	Genes related to synaptic plasticity and the myelin basic protein are dysregulated; some effects persisted into the subsequent generation.	27729240	Vassoler et al., 2017 [40]

## Data Availability

Not applicable.
